# The Plague

**Published:** 1910-11-05

**Authors:** Stephen Paget

**Affiliations:** Hon. Secretary, Research Defence Society. 21 Ladbroke Square, W.


### Editor's Letter-Box.

### THE PLAGUE.

*To the Editor of* The Hospital.

Sir,—In connection with the cases of plague in Suffolk, let me say that this Society lately published an illustrated pamphlet on "Plague in India, Past and Present," by Lieut.-Colonel Bannerman, M.D., D.Sc., Director of the Bombay Bacteriological Laboratory. It gives a full account of the experiments, which proved that fleas carry the plague from rats to man; it also gives a full account of Haffkine's preventive treatment, and of the many thousands of lives which have been saved by this treatment. I am sorry that the Research Defence Society cannot afford to give this pamphlet away in large quantities, but I shall be happy to send it to any of your readers who will send me seven stamps. I shall also be happy to send copies, on sale or return, to all booksellers.

I am, sir, your obedient servant,

Stephen Paget,

Hon. Secretary, Research Defence Society.

21 Ladbroke Square, W.: October 31.

